# 

**Published:** 1915-03-27

**Authors:** 


### CONTENTS.

PAGE

**The Army and the Medical Profession...** ... 567

**Guardians and the Demand for Nurses** ... 568

**Hospital and Institutional News ...** ... ... ... 569

The 1915 "Burdett"—A Plea for the Mobilisation of Hospitals and Nurses—The First Municipal Maternity Home—Mr. Samuel on Bradford's Child Welfare Schemes—A Great Medical Officer of Health—A Nonagenarian Chairman—The Attack on the Schools—The Epping Guardians and their Medical Officer—Clinical Assistants in an Infirmary—Ancoats Hospital, Manchester—Leicester as a Hospital Centre—Stealing X-Ray Apparatus—The Fishing Industry and the Hospitals—The Modern Coroner's Court—A Rest Cure at the Front—The Extension of Hertford County Hospital—The Value of the Honorary Secretary—Disabled Men in the New Army—German Trade-Marks for Drugs—Abolishing a Hospital's Constitution for the Duration of the War—The Leicester Board of Guardians and the Sanitary Committee—This Week's Drug Market—To Our Readers.

PAGE

**Massage and Mechano-Therapeutics at the Front ...** ... ... ... ... ... ... 575

The Importance of Hastening Convalescence.

**School Medical Inspection and the War** ... 576

The Part of the Medical Inspector.

**The Public Health of London** ... ... ... 577

The Medical Work of the Education Department.

**Restricting Admissions to Poor-Law Infirmaries** ... ... ... ... ... ... ... 578

**The Contract System in War-Time** ... ... 579

How it is Working and the Future Outlook.

**The Evolution of the Modern Hospital** ... 580

IV.—Medical Education, Administration, and Finance (*continued*).

**Medical Appointments** ... ... ... ... 581

**Round the World** ... ... ... ... ... 582

Fellow-Workers and the Roll of Honour.

**National Insurance Act Developments** ... ... 583

The New Amendment Act.

**Remarkable Hospital Chapels** ... ... ... 584

The Chapel of St. Luke the Physician, Camberwell House.

[*See over.*]

### CONTENTS—(*continued*).

| | PAGE |
|---|---|
| **The Editor's Letter-Box** ... ... ... ... | 585 |
| Hospitals and Payment for Wounded. | |
| **Institutional Needs** ... ... ... ... ... | 585 |
| **Maternity and Mothering** ... ... ... ... | 586 |
| The Case for Day Nurseries. | |
| **London Panel Committee** ... ... ... ... | 588 |
| **Buildings Contemplated** ... ... ... ... | 588 |
| **Obituary** ... ... ... ... ... ... ... ... ... | 588 |
| **The Institutional Worker** ... ... (Suppl.) | 1 |
| Institutional Workers and their Work: II. The Assistant Secretary. | |
| Question for March. | |
| Questions Invited. | |
| Employment and Training. | |
| Miscellaneous. | |

